# Kala-azar in Pregnancy in Mymensingh, Bangladesh: A Social Autopsy

**DOI:** 10.1371/journal.pntd.0002710

**Published:** 2014-05-01

**Authors:** Kazi Mizanur Rahman, Anna Olsen, David Harley, Colin D. Butler, Dinesh Mondal, Stephen P. Luby, Adrian C. Sleigh

**Affiliations:** 1 International Centre for Diarrhoeal Disease Research, Bangladesh, Dhaka, Bangladesh; 2 Australian National University, Canberra, Australian Capital Territory, Australia; 3 University of New South Wales, Sydney, New South Wales, Australia; 4 University of Canberra, Canberra, Australian Capital Territory, Australia; 5 Stanford University, Stanford, California, United States of America; New York University, United States of America

“She begged pardon from us, from her child, from Allah…while she was getting down from the bed she closed her eyes, and started moaning. When we held her and made her sit on the bed, she said…Mother, I am dying, I am dying. She collapsed on my lap. We splashed water on her face. We said Doa Durud [religious verse]…then phoned my son.” This is how the mother-in-law of the deceased woman depicted the moments around the death that happened in Fulbaria subdistrict of Mymensingh district, Bangladesh in 2011. During a large study on kala-azar or visceral leishmaniasis, we learned of this pregnant woman with kala-azar, who had recently delivered a stillborn infant and died one week after delivery. In this article, we report a social autopsy of this mother-child death from kala-azar in pregnancy in Bangladesh.

## Introduction

Kala-azar, meaning “black-fever,” is the local term in South Asia for visceral leishmaniasis. Kala-azar is targeted for elimination from South Asia by 2015. The control programme aims to reduce the annual incidence to less than one per 10,000 people [1]. The disease is usually fatal if untreated [1], and the case fatality rate is higher among women than men [2], [3]. Kala-azar among pregnant women places the foetus and mother at high risk of fatal outcomes [4], [5]. Moreover, women experience longer delays than men in seeking care and treatment for kala-azar, a problem well documented in Bangladesh [2]. Few studies have explored the personal and social dimensions of kala-azar, and even fewer have addressed the issues faced by women in pregnancy [2].

Using a mixture of methods and data sources, social autopsies review the psychosocial, economic, ecological, geographic, and health system factors associated with adverse health outcomes, including mortality [6]. In this study, for the social autopsy, we used a qualitative focus group discussion (FGD) involving next-of-kin of the deceased woman, including members of her household and neighbouring households. An interview of her husband was also conducted, using a pretested structured questionnaire.

Before any data collection, informed written consent was obtained from the participants. The participants (next-of-kin of the deceased woman) also gave consent for dissemination of the study findings and future use of the collected information. For illiterate interviewees, the consent form was read out loud and the participants' fingerprints were obtained on the consent forms as indication of their agreement to participate. Ethical approvals were provided by the Human Research Ethics Committee of the Australian National University and the Ethical Review Committee of the International Centre for Diarrhoeal Disease Research, Bangladesh (icddr,b). Both the ethics committees specifically approved the use of thumbprint procedures.

## Case Description

The woman was 35 years of age when she died. She was illiterate, with no formal education. She had been married for 10 years and was the second wife. The woman had previously had one child, aged under five years. Her husband, a businessman, also illiterate and with no formal education, was the head of the household. They owned the land on which their dirt-floor house was built and also additional cultivable land. They were from Fulbaria subdistrict of Mymensingh district, situated around 120 km northwest of Dhaka, the capital of Bangladesh. Fulbaria had the highest notified incidence of kala-azar in Bangladesh in 2011 [7].

In April 2011 the woman began to experience fever and developed abdominal distension and pain. She lost her appetite, and her body weight dropped. During her illness, she was jaundiced and had bleeding manifestations. She was pregnant with unknown gestation.

Initially the woman's family consulted a village doctor, an allopathic health care provider with limited training, based in their locality. As they said, “We went to him [the village doctor] first. He is from the village…we thought that it was a normal fever that would go away after one or two paracetamol or cotrim (co-trimoxazole) tablets.” The fever did not subside, and the woman was taken by her family to a qualified doctor based at the Fulbaria subdistrict health complex that was known and trusted by the family. According to them:

“[The qualified doctor] is a good doctor. We never had to visit [the qualified doctor] more than once [for any particular illness]. [This time] Allah's blessing did not work…We have belief in [the qualified doctor]. If someone says that he or she is poor [the qualified doctor] will not take any consultation fee. He is a good man.”

Initially the qualified doctor prescribed her “oral medications.” But after two visits her condition had not improved, and the doctor advised blood tests. On the basis of these blood test results, the doctor made a diagnosis of typhoid fever, and then prescribed daily intravenous ceftriaxone antibiotic injections for seven days, with which the fever subsided.

Two months later, the woman's symptoms recurred, including her fever. This time the patient was not taken to the same qualified doctor. According to the patient's family:

“How can we go back to [the qualified doctor] when the fever was not getting better after three visits…when the fever subsided but reverted back I [the husband of the deceased woman] said that this doctor would not do. We then went to the *Bideshi* [foreign] hospital after we became scared.”

This “foreign” hospital in Fulbaria was established and run by the internationally based nongovernmental organization (NGO) Médecins Sans Frontières (MSF) in collaboration with the Government of Bangladesh. The MSF facility was specialized in diagnosing kala-azar and providing free treatment using liposomal amphotericin-B, the safest and most effective medication for treating kala-azar, including in pregnancy [8].

At the MSF facility kala-azar was diagnosed based on clinical signs, history, and positive rK-39 test. Also noted was a haemoglobin level of 3.5 g/dl, far below the normal cutoff of 11 g/dl [9]. Because of the severity of the anaemia, the MSF health care providers advised the family to correct it before starting kala-azar treatment and referred the patient to a tertiary level hospital 30 km away from Fulbaria town, Mymensingh Medical College Hospital (MMCH), for blood transfusion. The family members had noticed the woman's pale hands and feet and understood the MSF doctor's instruction to seek blood. According to them, “She had no blood in the body.” From this point, the patient and her family started a new journey—a quest for a safe blood transfusion. It was then September in 2011, the end of the monsoon and five months after fever onset.

Each of the trips made to the Fulbaria town took approximately 2.5 hours each way. The family was grateful to the MSF clinic because travel costs were reimbursed by that facility. Adding to the hardship of travelling while ill, the area experienced severe flooding. The roads were submerged, slippery and treacherous. At times, the patient had to be carried on the shoulders of her family members.

“It was a very bad time; the area was inundated by flood… Water was even on the road. So, we had to carry her on our shoulder… When the water subsided, the road was left very muddy… We had to hold her tight while she was walking in such places… She used to be breathless all these times”.

At the MMCH the patient's family struggled to locate fresh, safe blood for transfusion. The doctors said that purchased blood “would not work.” The family tried to find a compatible donor among family members and also contacted voluntary blood donation organizations in Mymensingh town. Finally, they obtained half a bag of blood, which was then transfused to the woman. She returned home and her family contacted the MSF facility to advise them of the transfusion. MSF caregivers informed the family that one bag of blood would not be sufficient and that she would need three bags of blood before being eligible for kala-azar treatment.

The patient's family continued to seek blood. Eventually, the woman went into labour and gave birth by normal delivery to a stillborn baby. Family members said that the baby appeared mature and they believed that the delivery took place at full term. Her family had hoped that the woman's condition would improve after giving birth. They thought that “having the baby in the womb made her weaker” and that “drugs did not work on the mother because of the baby.” However, the family also reported that the woman lost blood and also lost consciousness at one point during the delivery.

According to the family, the woman had not received any specific antenatal care. However, during the course of her care-seeking for fever her pregnancy status was assessed several times, both by physical examination, and blood and urine tests. The focus group discussion could not clarify if these tests merely confirmed that the woman was pregnant or whether there were any clinical examinations or tests conducted to assess the baby's condition.

After surviving the delivery the woman stayed at home; she did not receive further care and died a week later. On the day she died, her husband had gone to Mymensingh in the morning to look for blood. According to him: “Suddenly…[she] looked very sick. We had to stay at home with…[her]. But, only Allah knows what will happen when. Can we just stay at home? Don't we need to try?” On route to the market in search of blood, the husband received a phone call to advise him of his wife's death.

During the focus group discussion, the participants reported this to be the first case of kala-azar during pregnancy in that community. No one in the family had ever been diagnosed with kala-azar, and they were unfamiliar with its symptoms. The family had not participated in the awareness campaigns, had not attended any kala-azar educational meetings, nor had they received information on the disease from television, radio, textbook, or newspaper.

Overall, the family spent approximately 18,000 Bangladeshi Taka (BDT) (US$225) in caring for the woman. This is equivalent to two to three months of income in a typical household in this region of Bangladesh. The family required a loan and had to sell land.

## Case Discussion

This social autopsy illustrates the interplay of many complex factors that culminated in the death of a woman suffering from kala-azar during pregnancy in Bangladesh. While it would be easy to blame the health care system for her untimely death, this intimate and nuanced analysis reveals that many other factors were operating, including social geography, climate, expensive and difficult transportation, poverty, lack of education, and cultural practices. The health system was slow to respond to the woman's needs, but in part, this reflects the difficulties related to diagnosis, particularly the distinction from typhoid fever. In retrospect, given the details of presenting symptoms, clinical features, and rK-39 serological test result, we can be confident that she had kala-azar. Knowledge of all these circumstances enhances prospects of devising appropriate health system responses. Accordingly, we will discuss in some detail the key elements leading to the woman's death, as well as relevant information from reported research.

Several studies have investigated outcomes of kala-azar in pregnancy after treatment. In a retrospective study in Bangladesh, among 16 pregnant women with kala-azar visiting a hospital, 11 were found to have experienced miscarriage [5]. There were no maternal deaths reported. All the patients who miscarried were in the 16th to 22nd week of pregnancy [5]. In Sudan, miscarriages and perinatal deaths occurred among mothers treated with sodium antimony gluoconate [4]. Maternal deaths also occurred. On the other hand, no pregnancy loss, no vertical transmission of kala-azar, and no maternal deaths have yet been observed with treatment with Liposomal Amphotericin B [10]–[14]. Clinical decision making in kala-azar in pregnancy is difficult; treatment can harm the unborn baby, but a decision to withhold treatment can lead to vertical transmission of *Leishmania* [15] and also harm the pregnant mother. Also, late diagnosis of kala-azar during pregnancy can cause maternal consequences, including severe anaemia [15], [16], as occurred in the case we have reported.

Anaemia is common in Bangladesh, especially among women of reproductive age and particularly during pregnancy. In a pregnant woman, haemoglobin should not fall below 11 g/dl. A recent survey found that 40% of pregnant Bangladeshi women suffer from anaemia [17]. Anaemia is also common in kala-azar. In a study in Bihar, India, the mean haemoglobin concentration of 282 kala-azar patients was found to be 7.5 g/dl with a range of 4.4 to 12.5 [18]. Correction of anaemia through blood transfusion or concentrated packed cell volume is generally recommended for severe anaemia with haemoglobin less than 7 g/dl [19]. But the kala-azar management guidelines are unclear concerning the order and timing of treatment for infection-associated severe anaemia [20].

For a fever that persisted, our study patient family followed a culturally traditional care-seeking pattern [21]. The woman and her family sought help from the private health system at village level, from the government health system at subdistrict level (primary health care) and district level (tertiary health care), and from an international NGO (subdistrict level). The health system responded but failed at all levels. The government system failed to make the diagnosis, and other system components were unable to coordinate treatment. Typhoid is common in this area [22], and it is not surprising that the family doctor tested the patient for this disease first. However, the usual serological test (Widal) is not very useful in endemic areas; it frequently produces false positive results, sometimes resulting from other infections, including kala-azar [23]. The comorbidity involving kala-azar and anaemia and the constraints due to poverty combined to impede health system efficacy. The social geography of the woman and her family did not connect well with available health services. In addition to the above, two health system features were major contributors to her demise—the absence of readily available safe blood and lack of antenatal care.

## Implications of the Study

We have studied a fatal case of kala-azar in a pregnant woman in Bangladesh. The analysis revealed the interaction between folk response to febrile illness and a poorly integrated health system aiming to eliminate kala-azar as a public health problem by 2015. A number of system-level changes would improve the outcomes of kala-azar patients in Bangladesh, while remaining sensitive to and realistic about health care provision in resource-poor settings.

The health system needs to improve diagnosis and case management. Health care workers in endemic areas should be educated to suspect kala-azar in febrile patients. This will require application of training resources, provided budgetary support can be obtained. As well, primary health care doctors should be encouraged to use the rK-39 rapid diagnostic test, which is now available to detect kala-azar at that level. Also, some revision is needed in the national kala-azar management guidelines for kala-azar during pregnancy. At present Liposomal Amphotericin B is recommended for kala-azar in pregnant woman but is not yet fully available as part of the government service. Clinical and preventive services for anaemia also need to be improved. Blood transfusion remains out of reach for most rural Bangladeshis, although it should not be thought of as first-line treatment for anaemia. Indeed, the social autopsy presented here reveals that prevention, detection, and treatment of anaemia all need to improve at all levels of health care in Bangladesh. Finally, our report shows that awareness of kala-azar is limited in rural areas in the endemic zone and health promotion campaigns are needed. Policy makers need to redouble their efforts regarding community education for kala-azar detection and control.

**Learning Points**

- Kala-azar in pregnancy can be fatal for both the mother and the baby.
- Misdiagnosis of kala-azar as typhoid is common and delays kala-azar diagnosis. Improved confirmatory diagnosis for both typhoid and kala-azar at point of care is needed.
- Anaemia can complicate pregnancy among kala-azar patients. Guidelines for kala-azar treatment in pregnancy need to be revisited to clarify the optimal way to integrate anaemia treatment with kala-azar therapy.
- Awareness of kala-azar among the community members needs to increase.
- The national kala-azar control programme has to be responsive to the social setting and geographical, economic, and cultural factors interacting with the health system.
